# A suture anchor-based repair technique for type IV jersey finger injuries: a biomechanical investigation

**DOI:** 10.1038/s41598-023-30373-w

**Published:** 2023-03-01

**Authors:** Gabriel Halát, Lukas Leopold Negrin, Paul Lennart Hoppe, Ewald Unger, Thomas Koch, Lena Hirtler, Stefan Hajdu

**Affiliations:** 1 Department of Orthopedics and Trauma Surgery, Division of Trauma Surgery, Medical University of Vienna, Vienna, Austria; 2 Center for Medical Physics and Biomedical Engineering, Medical University of Vienna, Vienna, Austria; 3 Institute of Materials Science and Technology, Faculty of Mechanical and Industrial Engineering, TU Wien, Vienna, Austria; 4 Department of Anatomy, Center for Anatomy and Cell Biology, Medical University of Vienna, Vienna, Austria

**Keywords:** Preclinical research, Tendons, Fracture repair

## Abstract

The aim of this biomechanical investigation was to evaluate a repair technique for type IV FDP tendon avulsions using a suture anchor, addressing the bony and the tendinous aspect of this injury simultaneously. In 45 distal phalanges from human anatomical specimens the injury was simulated and repairs were performed with a suture anchor using an innovative technique, interosseous sutures and a combination of screws and an interosseous suture. Repetitive loading for 500 cycles simulated postoperative mobilization. Repairs were loaded to failure thereafter. Elongation of the tendon-suture complex, gap formation at the bone-bone contact line and at the bone-tendon insertion line, load at first noteworthy displacement (2 mm), load at failure and the mechanism of failure were assessed. The suture anchor technique was superior biomechanically considering load at failure (mean: 72.8 N), bony gap formation (mean: 0.1 mm) as well as tendinous gap formation (mean: 0.7 mm), implying a preferable stability of the repair. Overall, this study demonstrates good ex vivo mechanical stability for a proposed suture anchor repair technique for type IV FDP tendon avulsion injuries, which might enable early postoperative mobilization in patients. The technique's subcutaneous implant placement and low implant load are expected to reduce potential complications observed in other commonly used repair techniques. This approach warrants further evaluation in vivo.

## Introduction

In 1977, Leddy and Packer^1^ introduced a classification system for flexor digitorum profundus (FDP) tendon avulsion injuries consisting of three avulsion types. Each of the types is defined by the level of FDP tendon retraction and potential concomitant bony injury^1–5^. Smith^6^ later added a fourth type to this classification describing a bony fragment avulsion associated with a tendon separation from the fragment and retraction along the flexor tendon sheath to a variable degree. Among tendon avulsion injuries in the hand, the probability of encountering a type IV avulsion of the FDP tendon is rather low and recommendations for surgical treatment are limited, substantiated mainly by case reports. Techniques and technique combinations ranging from distal interphalangeal (DIP) joint arthrodesis, K-wire fixation, interosseous sutures, screw fixation, pull-out wires to miniplate fixation techniques were proposed^3,6–14^. Consistent with postoperative treatment algorithms of flexor tendon injuries in general, the goal of a satisfying functional outcome can only be achieved by a rigid repair construct enabling a reasonable balance between protection of the repair and prevention of adhesion formation during early postoperative mobilisation^15–17^.

Due to the nature of this injury, repairs demand the combination of techniques attaining a rigid fixation of the avulsion fragment, as well as a solid reattachment of the retracted tendon^1,18–21^. Infections, repair construct disintegrations and secondary dislocations with loss of joint congruity leading to posttraumatic DIP joint arthrosis have been described primarily in external fixations. In contrast, internal fixation techniques have proven to be less prone to fulminant complications in other types of FDP tendon avulsions, seeming favourable for the treatment of a type IV injury as well. Suture anchor-based techniques were introduced to facilitate internal fixation of tendons and recent findings in solitary tendon- or bony FDP tendon avulsion repair seem promising^10,18,22–33^. Adopting findings from our previous study^31^ using a suture anchor combined with an interosseous suture pattern based on a tension banding principle to a type IV injury, we believe to attain a biomechanically stable repair avoiding reported complications.

The aim of this investigation was to conduct a biomechanical evaluation of a suture anchor technique, hypothesizing a sufficient rigidity and stability during early mobilisation simulation. In order to outline the advantages of this internal repair technique, we compared the biomechanical characteristics of the suture anchor technique to a double suture interosseous fixation and a screw repair combined with a single suture interosseous tendon fixation.

## Material and methods

Although no specific biomechanical testing protocols for type IV FDP tendon avulsion were identified in the literature, a variety of procedures for injury simulation and biomechanical repair evaluation of other types of flexor tendon avulsions have been described^30,31,34–38^. Based on the methods described by our group in 2018^31^ we embraced a curvilinear testing model to examine biomechanical characteristics during cyclic loading (postoperative mobilisation simulation) and when loaded to failure^34,39^. None of the tissues used in this study were procured from prisoners. All specimens were obtained at the Center for Anatomy and Cell Biology, Medical University of Vienna where all of the body donors provided written informed consent prior to their death for use in teaching and science. The Institutional Ethics Committee of the Medical University of Vienna approved this study. All methods were performed in accordance with the relevant guidelines and regulations.

### Specimen preparation and repair

Fifteen fresh-frozen hand-specimens (seven female, eight male) were randomly selected (mean 75, range 65–85 years of age). The specimens were thawed at 4 °C for 48 h prior to dissection. Intending to avoid bias originating from increased loss of bone density due to particular diseases donor records were reviewed. No signs of impaired bone quality were recognized during specimen preparation. From these 15 hand specimens, 45 fresh-frozen distal phalanges with an intact FDP tendon attachment from the ring, middle, and index fingers were exarticulated at the DIP joint and separated from surrounding soft tissues. The designated tendon length between the volar base of the distal phalanx and the point of tendon transection was 12 cm. At the level of the A4 pulley we determined the cross-sectional area (CSA) of the tendon measuring the tendon diameter and calculating the circle area in mm^2^. To allow full visualisation and further evaluation of the repair during cyclic testing, adjacent soft tissues and the A5 pulley were excised. Injury was simulated by separating the tendon from its bony insertion with a scalpel and creating a volar base avulsion fragment including at least 30% of the articular line using a chisel. Rehydration of the specimen was ensured by storage in saline solution (0.9% NaCl) until testing. Throughout preparation and testing, specimens were irrigated with this solution periodically.

**Figure 1:**
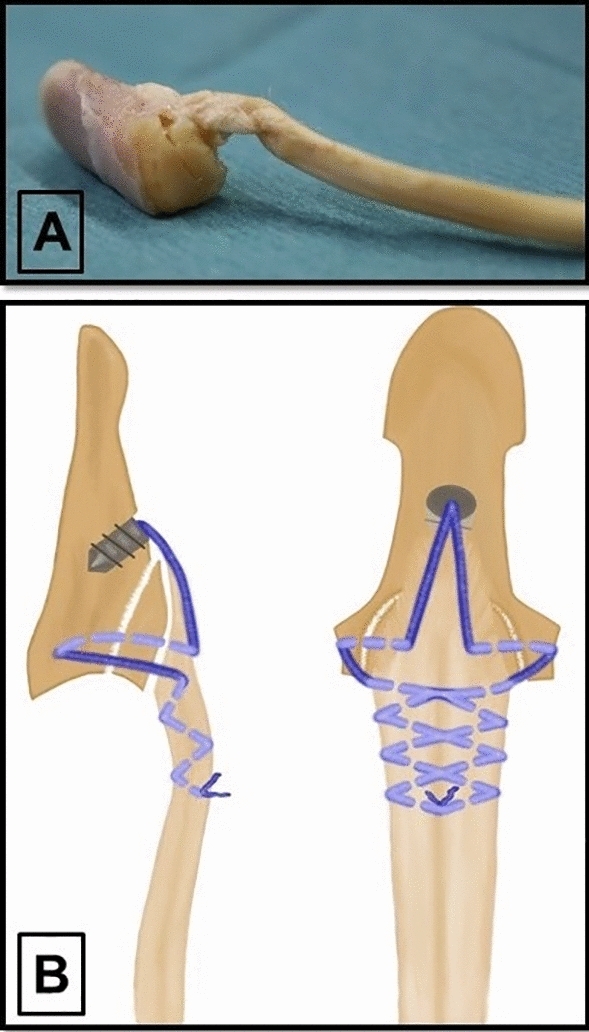
(**A**) Specimen repaired with the suture anchor technique. Articular line alignment is visualized. (**B**) Illustration of the suture anchor technique depicting implant position, as well as the pattern of suture placement within the distal phalanx, the bony avulsion fragment and the tendon.

Three study groups were formed by random allocation of 15 specimens to each group. In the first group (termed “anchor”), repair was conducted with a Corkscrew 2.2 × 4 mm suture anchor loaded with a #2-0 FiberWire (Arthrex, Naples, Florida) using the concept of an intratendineous and interosseous suture placement concordant with a novel repair technique presented in our previous study^31^ with the exception of addressing the complex injury characteristics of a type IV FDP tendon avulsion injury. This suture anchor technique follows a tension banding principle, increasing pressure on the avulsion fragment and the reattached tendon when strain is applied to the tendon and eventually the repair complex. The mean CSA of the FDP tendons was 6.1 ± 1.8 mm^2^. Intact cortical bone, approximately 1–2 mm distal to the bony avulsion site was considered the optimal anchor insertion area. A 45° retrograde insertion angle was chosen to reduce the probability of implant extrusion during loading of the repair^29,36^. After anchor placement, we guided each anchor suture through the distal stump of the tendon and subsequently inserted both sutures into the bony fragment at the borders between the median and the lateral thirds of its volar surface. Both sutures were then inserted into the corresponding site of the avulsion area of the distal phalanx. Following penetration of the dorsolateral cortex of the distal phalanx, sutures were retrieved on both sides of the distal phalanx. At this point, pulling on the sutures led to a reduction of the avulsion fragment and a reattachment of the tendon to its insertional footprint. We then inserted both sutures into the tendon in a transverse manner at the level of the volar base of the distal phalanx and tightly wove the sutures together along the median line of the FDP tendon. A Bunnell suture pattern was utilized to ensure a sufficient tendinous hold grasping approximately 1 cm of the distal tendon stump. A buried 5-throw square knot concluded the repair (Fig. 1).

**Figure 2:**
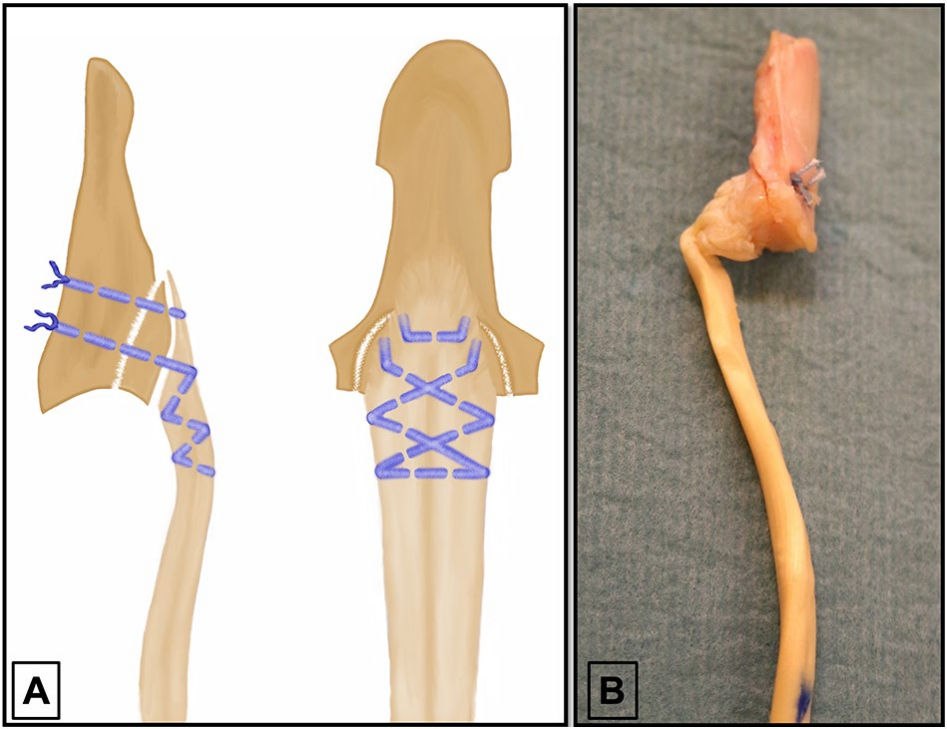
(**A**) Illustration of the interosseous suture reattachment of the bony and tendinous injury components. To allow a clear perception of the suture placement within the tendon and the avulsed bony fragment in the top view, dorsal aspects of the sutures are not depicted. (**B**) Photographic image of a specimen repaired with interosseous sutures.

We reattached both injury components using two serial interosseous sutures in the second group (termed “interosseous”). The suture material, a #2-0 FiberWire suture (Arthrex, Naples, Florida), equalled the suture material used in the “anchor” group. The distal interosseous suture grasped the distal end of the FDP tendon stump, held the distal aspect of the avulsion fragment in its avulsion footprint, and was guided out through the intact dorsal corticalis of the distal phalanx. Main reduction of the articular line and compression of the fragment was achieved with the proximal interosseous suture. First, this suture was woven into the distal tendon stump using a Bunnell suture pattern. Subsequently the threads were guided through the avulsion fragment as well as into the intact portion of the distal phalanx. A 5-throw square knot at the dorsum of the distal phalanx proximal to the germinal matrix finalized each of the two sutures (Fig. 2). Calculation of the CSA in this group resulted in a mean of 7.1 ± 2.3 mm^2^.

**Figure 3:**
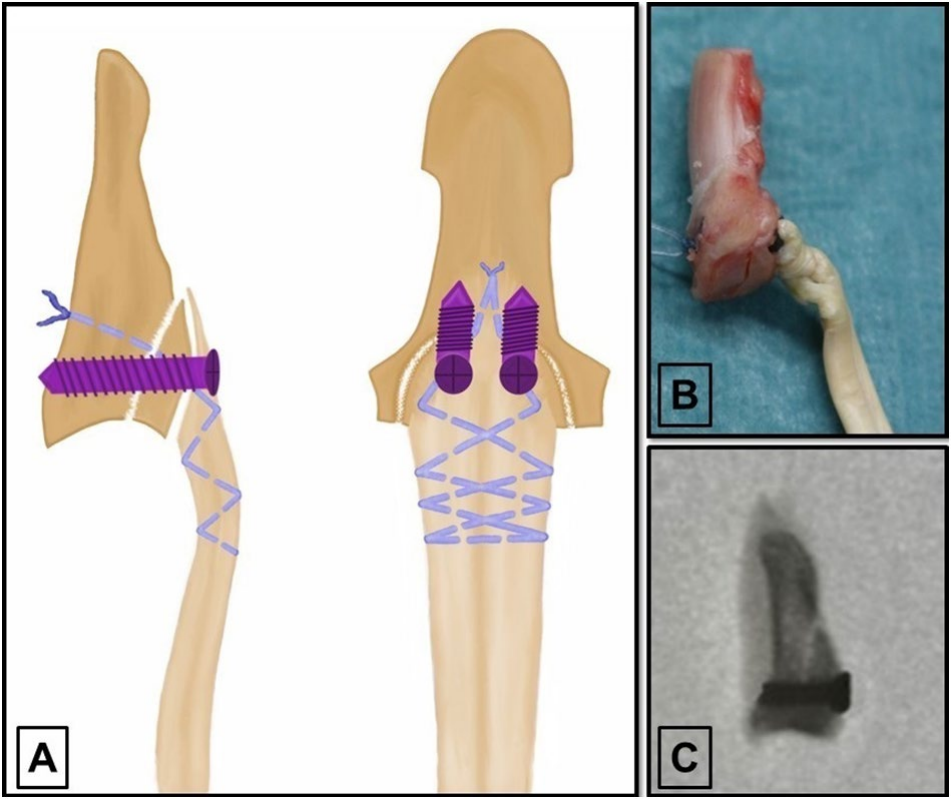
(**A**) Type IV injury repair using minifragment screws for bony avulsion repair and an additional interosseous suture for tendon reattachment. (**B**) Specimen after repair with screws and an interosseous suture. (**C**) Lateral fluoroscopy image of the distal phalanx after avulsion fragment repair with screws.

In the third group (termed “screw”) we repaired the simulated injury combining a double screw fixation of the avulsed fragment and a single interosseous suture reattachment of the tendon. Two cannulated cortical titanium screws 2.0 × 8 mm (Arthrex, Naples, Florida) were inserted after verification of good fragment reduction and an aligned articular line. A #2-0 FiberWire suture (Arthrex, Naples, Florida) was woven into the proximal tendon stump using a Bunnell suture pattern. Approximately 5 mm distal to the level of screw insertion the threads were inserted through the reduced avulsion fragment into the intact distal phalanx approximating the tendon stump to the avulsion fragment. The suture was secured with a 5-throw square knot at the dorsal aspect of the distal phalanx sparing the germinal matrix (Fig. 3). The mean CSA of the tendons repaired with screws was 6.6 ± 1.4 mm^2^.

### Data acquisition and testing

**Figure 4:**
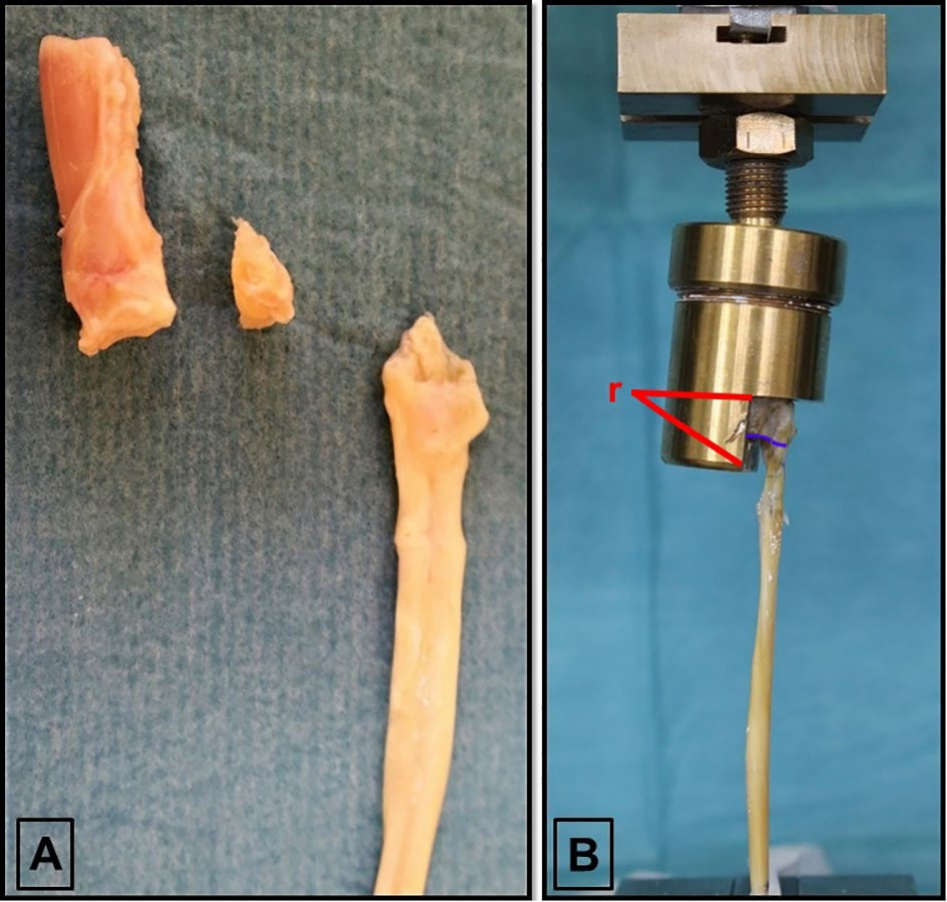
(**A**) Specimen preparation and injury simulation prior to repair. (**B**) Repaired specimen secured in the DP holding cylinder mounted on the tensile testing machine. Distance (r) indicating the 10 mm reference for further image evaluation.

The biomechanical testing setup and the data acquisition protocol were adopted from our previous investigation^31^ evaluating repair techniques of type III FDP tendon avulsion injuries, seeming most suitable for the investigation of biomechanical characteristics of type IV injury repairs as well. To establish a firm hold of the specimens and to enable visualisation of the dynamics of the repair throughout biomechanical testing we used a distal phalanx (DP) holding cylinder mounted on an electromechanical tensile testing machine (Zwick Z050, ZwickRoell GmbH, Ulm, Germany) equipped with a 1 kN load cell. Prior to testing the load cell was checked for its accuracy in the low load region using calibrated weights. In the range from 0.1 to 5 N the maximum deviation was 7 mN. Following repair, each specimen was placed into the DP cylinder allowing full exposure of the repair through its semicircumferential window. Using common white plaster as a fixation material, all repair components remained unaffected, thus preventing unintended rotation or tilt of the specimen within the cylinder. A ball-joint linking the tensile testing machine to the cylinder ensured an optimal alignment of the specimen-repair construct along the longitudinal axis of the distal phalanx and the reattached tendon when strain was applied, preventing bias by translational forces to the repair site. Seven centimetres proximal to the repair, the tendon was retained using a standard tensile clamp. The articular line of the distal phalanx, of the avulsion fragment, as well as the tendon at the corresponding articular line level were marked with a blue felt tip pen under a baseline preload of 2 N allowing visual assessment of the repaired injury components (Fig. 4). Continuous cyclic loading from 2 to 15 N at a rate of 5 N/s for a total of 500 cycles simulated immediate postoperative mobilisation. Upon completion of cyclic loading, specimens were loaded to failure at a rate of 20 mm/min. A high-resolution camera placed in front of the experimental setup documented bony and tendinous gap formation at the repair site at the initial 2 N preload, periodically every 100 cycles, as well as, when loaded to failure, disclosing failure mechanisms. Consistent with methods used in our former studies^30,31^ on FDP tendon avulsion repair, the load at first noteworthy displacement (2 mm) representing the load at the occurrence of an avulsion fragment- or reattached tendon derived, clinically relevant global system displacement was evaluated. To allow a thorough perception of the repair- and failure biomechanics, following data were recorded and analysed as well: load at failure, elongation of the system, gap formation at the bone–bone contact line (proximalisation of the fragment resulting in an articular step off), gap formation at the bone–tendon insertion line, and the mechanism of failure. Reaching a structural balance of the tendon and the suture material after 50 cycles, elongation of the system was measured between the 50th and the 500th cycle. Bony or tendinous dynamics at the repair site documented on photographs were analysed with Image J Software (National Institutes of Health, Washington, DC, United States).

### Statistical analysis

Statistical analysis was performed using IBM SPSS Statistics Version 26, 64-bit. Normal distribution was assessed for every parameter by the analysis of histograms. Subsequently, for normally distributed data, one-way analysis of variance (ANOVA) was performed to detect any differences between the three repair techniques. Homogeneity of variance was given in every parameter; therefore, the Tukey post-hoc analysis for pairwise comparison was used. Analogously, for non-normally distributed data, the Kruskal–Wallis test and the Dunn´s post-hoc test were utilized. Data of normally distributed parameters are presented as mean and standard deviation, non-normally distributed data as median and interquartile range. p-values lower than 0.05 were considered statistically significant.

## Results

**Figure 5:**
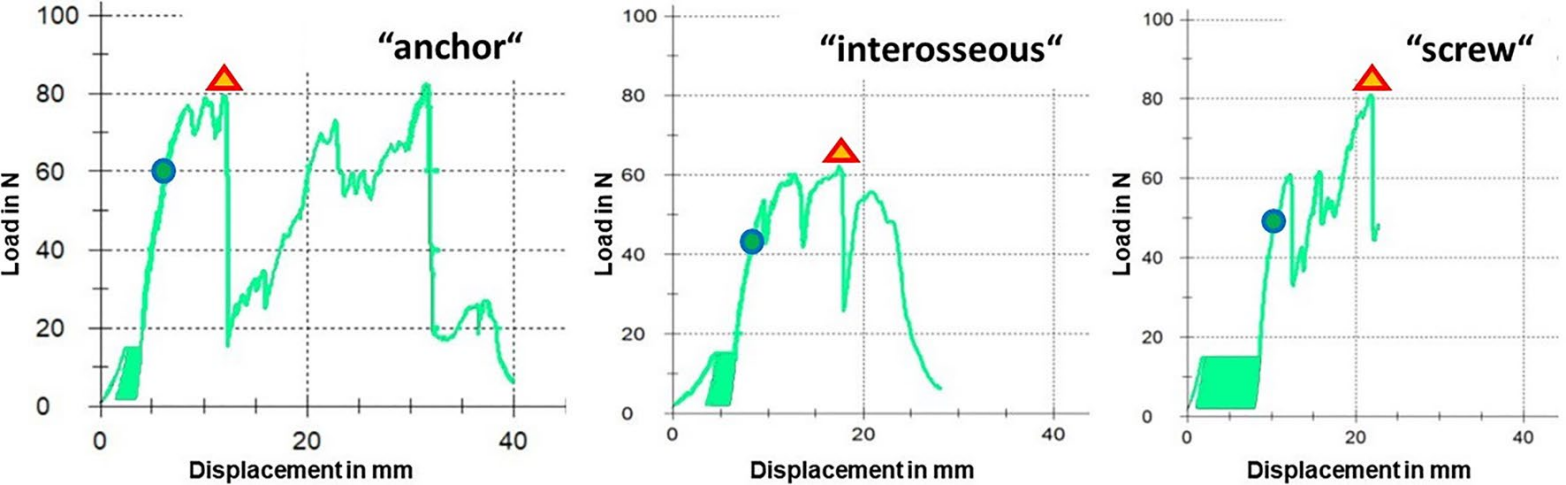
Exemplary load versus displacement curve-graphs for each particular repair technique. The circle marks the point of measurement of the “load at the first noteworthy displacement (2 mm)”. The triangle indicates the “load at failure” assessment point.

We were able to simulate the injury in each specimen in a consistent manner. When performing the repairs, no adverse events emerged resulting either from the repair procedure or from structural quality of the specimens. The biomechanical setup fulfilled our expectations on a rigid hold of the distal phalanx and a firm capture of the proximal tendon stump. Regarding the homogeneity in tendon size, we observed no statistically significant difference in CSA of the tendons between our study groups (p = 0.30). During cyclic loading no distinct failures occurred at the repair site such as extrusion of the distal phalanx out of the DP cylinder, premature implant tear-out, suture cut-out or tendon rupture, nor did we encounter any technical failure of the testing machine. In the course of each biomechanical testing, elongation was the first parameter to be evaluated. A displacement versus force curve was generated for each specimen enabling determination of elongation and potential displacement range throughout cyclic loading (Fig. 5). A statistically significant difference of elongation between all three study groups was detected in favour of the suture anchor repair technique (p = 0.001). The indicated median elongation in the “anchor” group was 0.79 (IQR 0.70–1.27) mm, 1.03 (IQR 0.74–1.27) mm in the “interosseous” group, and 1.6 (IQR 1.07–2.26) mm in the “screw” group, respectively. Photographic image analysis after 500 cycles revealed a mean gap formation at the bone-bone contact line of 0.1 ± 0.06 mm in the “anchor” group compared to 1.0 ± 0.41 mm in the “interosseous” group and 0.2 ± 0.22 mm in the “screw” group. The least bony gap formation was found in the “anchor” group (p =  < 0.001). Nevertheless, extent of bony gap formation between the “anchor” group and the “screw” group was not statistically significant (p = 0.580). At the bone-tendon insertion line, the least gap formation was observed using the suture anchor repair technique as well (p < 0.001). The measured bone-tendon gap was 0.68 ± 0.24 mm in the “anchor” group, 1.97 ± 0.57 mm in the “interosseous” group and 1.5 ± 0.61 mm in the “screw” group. By the time repairs were loaded to failure, the strain on the tendon increased and the load at first noteworthy displacement (2 mm) was captured on the displacement versus force curve. No statistically significant difference (p = 0.311) was observed between our three study groups (Table 1). While the strain reached a critical magnitude, we observed the failure to be a sequence of close events beginning with minimal negative spikes as a sign of suture material breakage and subsequently disintegration of the repair. Statistically significant higher load at failure values indicated the superiority of the suture anchor repair technique in load tolerance (p = 0.004). Repairs failed either by anchor pull-out from the bone or suture cut-out within the tendon when the suture anchor technique was applied. Interosseous suture repairs expectedly failed by suture cut-out in most cases, interestingly a fracture of the distal phalanx occurred as the mechanism of failure in 5 specimens. In the “screw” group, the failure mechanisms were either a screw pull-out or suture cut-out (Table 2).

**Table 1:** Tension force values of the repair techniques.

	Anchor (n = 15)	Interosseous (n = 15)	Screw (n = 15)
Load at first noteworth. displ. (N) mean ± SD	36.97 ± 5.5	39.74 ± 6.8	36.41 ± 6.4
Load at failure (N) mean ± SD	72.77 ± 15	54.17 ± 20.9^a^	51.07 ± 7.4^a^

^a^Statistically significant difference compared to the “anchor” repair technique.

**Table 2:** Mechanisms of failure after continuous increase of strain.

	Anchor (n = 15)	Interosseous (n = 15)	Screw (n = 15)
Screw/anchor pull-out	3	N/A	2
Suture cut-out	12	10	13
Distal phalanx fracture	–	5	–

## Discussion

A considerable variety of repairs for type IV FDP tendon avulsions has been introduced so far. Primarily case reports on diagnostic approaches, as well as repair techniques in particular were identified in the literature^7–13^. No biomechanical studies on type IV injury repairs, nor clinical reports on suture anchor repairs of this injury were identified in the present literature. Reported repairs mainly based on K-wire or screw fixation of bony avulsion fragments and pull-out suture reattachment of the avulsed tendon. Each of these repair techniques holds a significant risk of complications and when combined, the incidence of complications may further increase^1,18–20,32,34^. Conclusions on therapy related outcomes are limited by omitted postoperative protocols and scarce consistency on outcome reports. Considering the findings of Leversedge et al.^40^ tendon nutrition may be impaired in a severely retracted tendon requiring timely repair. Therefore, we advocate additional imaging by ultrasound or MRI in each injury with a volar avulsion fragment of the distal phalanx.

The aim of this study was to introduce and biomechanically validate a firm and rigid repair technique using a single implant addressing both injury components; the bony fragment and the avulsed tendon. Moreover, an internal fixation technique is expected to result in a low incidence of peri- and postoperative complications, thus increasing the probability of a satisfying functional outcome. In recent literature, suture anchors moved into the focus of tendon repairs of the hand due to improved materials with a high load tolerance despite the small implant size^18,22,23,30,34,41,42^. A suture anchor based technique was already introduced for a type III injury repair^31^. The application appeared to be independent of avulsion fragment size, therefore supposedly preventing complications such as fragment breakage due to drilling or overtightening of screws. The superiority of the repairs’ biomechanical characteristics led us to the inquiry, weather it may be suitable for a type IV tendon avulsion as well.

The lower elongation in the “anchor” group reflects the formation of a solid conjunction of both injury components acting as a single avulsion element. This may imply a positive effect during postoperative mobilisation, as strain forces seem to be distributed equally between all affected structures. We divided the overall gap formation into two distinct parameters; gap formation at the bony avulsion site and at the bone-tendon insertion. Thus, we were able to draw conclusions on repair characteristics comparing the biomechanical data between study groups. Eglseder et al.^8^, as well as Trumble et al.^13^ used a single minifragment screw for a type IV injury repair. To improve fragment compression and ensure rotational stability, we decided to establish a double screw repair. Supporting our hypothesis of the suture anchor repair technique’s rigidity, we did not detect a statistically significant difference of bony gap formation compared to the screw repair. The tension banding principle may be the primary cause for this finding. Considering the material properties of the sutures used and application of a comparable intratendinous suture pattern in all three study groups, we expected a similar gap formation at the bone-tendon insertion after 500 loading cycles. Interestingly, our observation revealed a superiority of the anchor technique. We believe the same sutures grasping the tendon twice and tightening it to the bone to be the most probable rationale. This parameter did not show any statistically significant difference between screw- and interosseous repairs, although the tendinous gap formation was the largest in the interosseous group. Highest load at the first noteworthy displacement was measured in the “interosseous” group. The suture anchor technique, as well as screw repairs seem to provide the most rigid hold in the lower loads region emphasizing their relevance during assisted postoperative mobilisation. Repair failure occurred at significantly higher loads in specimens treated with the suture anchor technique.

Concordant with our prior findings^31^ evaluating a suture anchor technique to repair a type III FDP tendon avulsion, we found the load at failure to be the highest and the gap formation to be the lowest when the same technique was applied. We previously observed a mean load of 100.5 N, whereas in this study a mean load of 72.7 N was detected when the anchor technique was loaded to failure. We believe that this difference originates from the injury composition, requiring the repair to stabilize not only a bony avulsion but a bony fragment and an avulsed tendon simultaneously. The tension banding principle of the suture pattern seems to be equally effective in preventing relevant bony gap formation in both injury types. In our prior investigation of type I injuries^30^, we observed mean failure loads of 65 N using an identical implant, securing the tendon to the bone applying a Bunnell suture pattern only. In general, due to increased resistance to tendon gliding resulting from postoperative soft tissue edema, intratendinous flexor tendon sutures are believed to require an initial strength of at least 30–55 N to allow gentle or moderate active finger motion^43–48^. The combination of the chosen implant, the suture material and the suture pattern in this study seems to outbalance the load tolerance of other distinct techniques addressing each injury component separately, underlining the biomechanical superiority of the here proposed technique.

### Limitations

Extracting the tendon out of the flexor tendon sheath and exarticulating the distal phalanx, as well as the age of the used specimens and their thawing are relevant limitations of our ex vivo study design. Screws with a diameter of 2 mm were the thinnest available. In clinical use, a screw diameter of 1.5 mm or 1.8 mm would be preferable, although we did not observe any avulsion fragment disintegration or distal phalanx fracture during repair or cyclic loading. Evaluation of load at first noteworthy displacement was based on a global system displacement measurement. To enhance data quality of this critical parameter, local displacement measurement would be preferable in forthcoming investigations. Assessment of repair dynamics under more physiologic circumstances, with the flexor tendon sheath preserved seems to be the most reasonable next step in future biomechanical investigations. To understand the tendency towards potential complications that cannot be anticipated in an ex vivo biomechanical study subsequent clinical trials will be essential.

## Conclusion

In this first biomechanical study on type IV jersey finger repairs we validated the here presented testing setup and were able to underline the superior biomechanical characteristics of a suture anchor-based repair technique. Our findings indicate a potential benefit for the treatment of this challenging injury when applying our single implant solution due to a low probability of peri- and postoperative complications and sufficient load tolerance. However, future clinical investigations will disclose the suture anchor repair’s behavior in vivo throughout the healing process, thus revealing its relevance in surgical practice.

## Data Availability

The datasets generated during and/or analysed during the current study are available from the corresponding author on reasonable request.
